# Alkaptonuria with rapidly destructive arthropathy of the hip: A case report and literature review

**DOI:** 10.5152/j.aott.2021.21205

**Published:** 2021-11-01

**Authors:** Yoshiki Kitahara, Nobuhiro Kaku, Hiroaki Tagomori, Hiroshi Tsumura

**Affiliations:** Department of Orthopedic Surgery, Oita University, School of Medicine, Yufu City, Oita 879-5593, Japan

**Keywords:** AlkaptonuriaHomogentisic acidDestructive hip arthropathy

## Abstract

Alkaptonuria-related rapidly destructive arthropathy of the hip joint has not been reported in detail with both imaging and histopathological findings in the literature. We, herein, presented the case of a 79-year-old male patient who suddenly started experiencing marked right hip pain. Radiography showed that the femoral head was spherical; however, after 3 months, approximately half of the femoral head was destroyed despite there being almost no change in the acetabulum. Radiographs of the spine also showed fusion between multiple vertebrae. Significant osteoporosis was observed on roentgenography, together with decreased bone density. Urinary gas chromatography-mass spectrometry analysis revealed that a large amount of homogentisic acid was excreted. During total hip arthroplasty, gray and muddy contents were observed in the joint capsule, and the surface of the destroyed femoral head was black. Histopathologically, granulomatous foci containing fragmented bone and cartilage debris were found in the bone marrow space of the joint surface, and the cartilage tissue was pigmented brownish black. The patient was subsequently diagnosed with ochronotic hip joint destruction. The present case report is the first to demonstrate rapidly destructive coxopathy associated with alkaptonuria using both imaging and histopathological findings. These findings clearly show that severe hip joint destruction defined as rapidly destructive hip arthropathy can occur in a very short time period for patients with alkaptonuria.

## Introduction

Alkaptonuria develops due to abnormal metabolism of phenylalanine and tyrosine due to homogentisic acid (HGA) oxidase deficiency, which causes HGA accumulation. It has an incidence of 1 in 250,000–1,000,000 live births.^1^ Alkaptonuria is diagnosed based on a high level of urinary HGA and a urine color change that occurs over time. The disease clinically presents as black discoloration of the urine and bluish-black discoloration of connective tissues; the latter is known as ochronosis. Alkaptonuria causes arthropathic changes in the large joints, such as the shoulders, knees, and hips, due to pigmentation of the soft tissues, especially the cartilage, ligaments, and synovium, which in certain cases require surgical treatment.^2,3^ Rapidly destructive arthropathy has been defined as femoral head destruction occurring within 6 months. It is not a single disease entity,^4^ but rather a collective term encompassing rapid joint destruction due to various causes, compromising bone structure. Even primary osteoarthritis, avascular necrosis, and rheumatoid arthritis may lead to rapid destruction. There is no report that clarifies typical rapidly destructive coxopathy (RDC) associated with alkaptonuria using both appropriate imaging and histopathological findings. We present the case of a patient with RDC associated with alkaptonuria.

## Case Presentation

**Figure 1.**
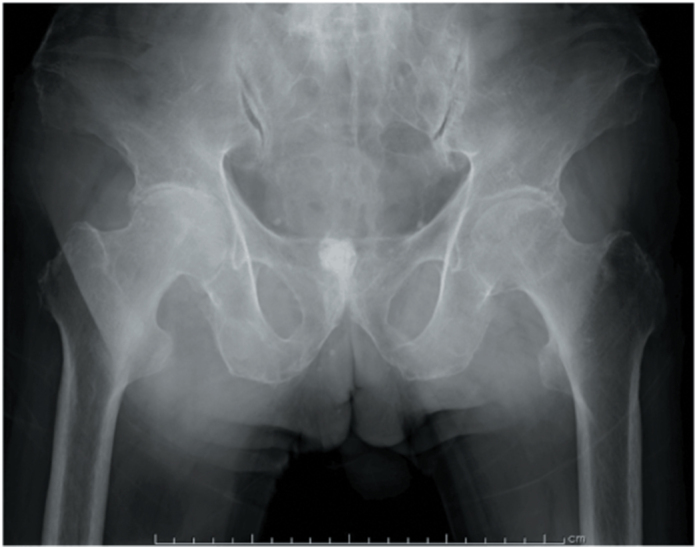
An anteroposterior radiograph of the hip joint at the first visit. A slight narrowing is observed in the right joint space, although the femoral head remains spherical. Other findings of arthropathy are also scarce. The sacroiliac joint is not fused.

A 79-year-old man experienced severe acute pain in his right groin while performing his daily activities. There was no history of trauma, such as a fall. He was admitted and treated with analgesics and bed rest. Hip joint roentgenography showed only slight joint space narrowing and no morphological abnormalities of the bone; there were no osteophytes or cysts on the acetabulum or femoral head, and the femoral head remained spherical (Figure 1). He was transferred to our hospital because his right hip pain did not resolve after 3 months.

Additionally, he had no history of any joint diseases that affected his musculoskeletal condition, such as rheumatoid arthritis. Although he recalled having black urine stains on his underwear during childhood, he never divulged this to others or sought treatment. No family members had any known metabolic abnormalities.

Upon admission, his height, weight, and body mass index were 1.58 m, 52 kg, and 20.8 kg/m,^2^ respectively. Furthermore, he had a normal level of intelligence and body temperature, and he experienced no morning stiffness or significant swelling or pain in other joints. However, he experienced slight pain in both knees. Bilaterally, his auricles were partially pigmented.

At admission, he was unable to walk unaided. Although he experienced neuropathic pain in the right hip during movement without resting, and despite marked tenderness in the Scarpa's triangle, no redness or swelling was observed. The affected hip joint range of motion was reduced by about half compared to that on the contralateral side. The difference in leg length was about 10 mm (right < left), measured between the anterior superior iliac spines and the medial malleoli, and his modified Harris Hip Score was 22.

**Figure 2.**
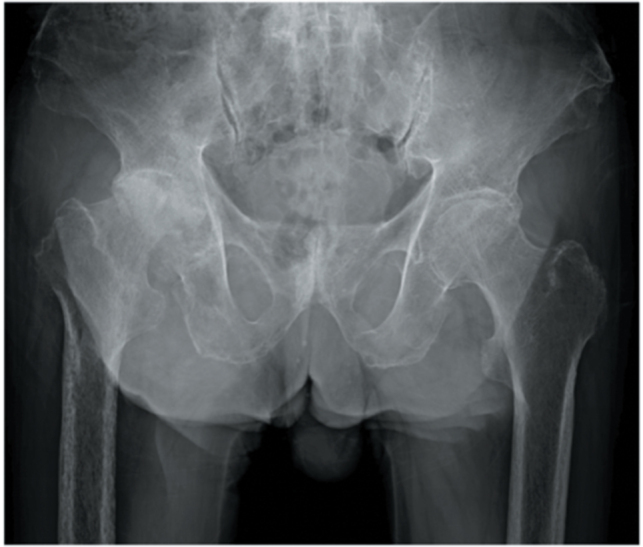
An anteroposterior radiograph of the hip joint after 3 months. The right femoral head is approximately halved and the joint space has disappeared, although the acetabulum is less destroyed compared to the changes in the femoral head.

**Figure 3.**
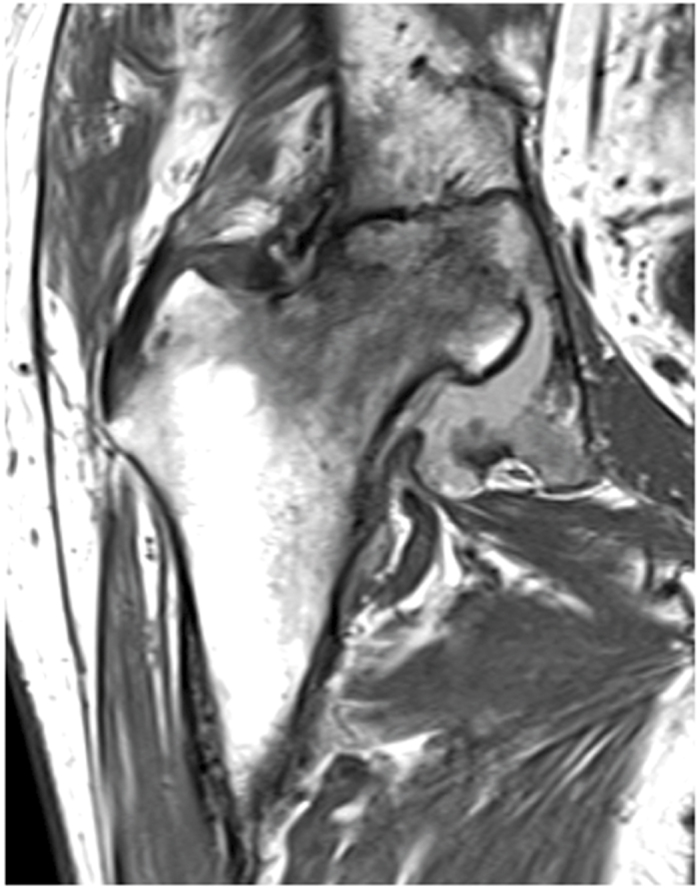
T1-weighted magnetic resonance imaging.

**Figure 4.**
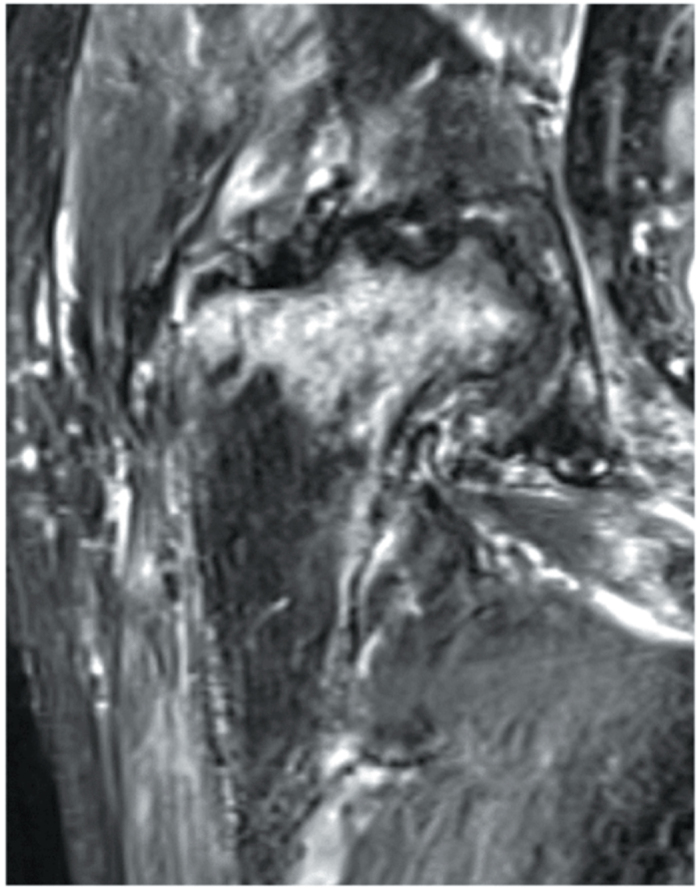
T2-weighted magnetic resonance imaging. On the weight-bearing part of the femoral head, there is a band-shaped hyperintense and isointense region on T1 imaging and a hypointense region on T2 imaging. In the area extending from the femoral head to the neck, there is a hypointense region with an unclear boundary on T1 imaging and a hyperintense region on T2 imaging. Magnetic resonance imaging reveals luminance changes that show bone edema, while bone defects are also observed on the acetabular side.

Although roentgenography after 3 months showed almost no change in the acetabulum, the femoral head was destroyed, and approximately half of the head had been eroded (Figure 2). The sacroiliac joint had mild osteosclerosis but was not fused. Moreover, increased bone radiolucency was observed, likely due to disuse atrophy. Magnetic resonance imaging revealed a band-like region at the weight-bearing surface of the femoral head, which was hyper- and isointense on T1-weighted images and hypointense on T2-weighted images. Furthermore, T1-weighted images revealed a hypointense area with an unclear boundary at the distal end of the femoral head. On T2-weighted images, the hyperintense area extended to the femoral neck (Figure 3 and Figure 4). Lumbar spine roentgenography showed high radiolucency and narrowing, calcification, and fusion of the vertebral bodies (Supplementary Figure S1).

The bone mineral density of the lumbar spine and left femoral neck were 0.487 g/cm^2^ and 0.431 g/cm,^2^ respectively.

Laboratory findings revealed a C-reactive protein concentration of 0.17 mg/dL, while tests for rheumatoid factor and anti-cyclic citrullinated peptide antibodies were negative. Other hematological and biochemical tests revealed no abnormalities. The calcium and phosphorus concentrations were 9.27 mg/dL and 3.48 mg/dL, respectively. The concentration of tartrate-resistant acid phosphatase 5b (TRACP-5b) was 886 mU/dL. His urine turned brown when exposed to air for 24 hours. Urinary gas chromatography-mass spectrometry analysis revealed that a large amount of HGA was excreted.

**Figure 5.**
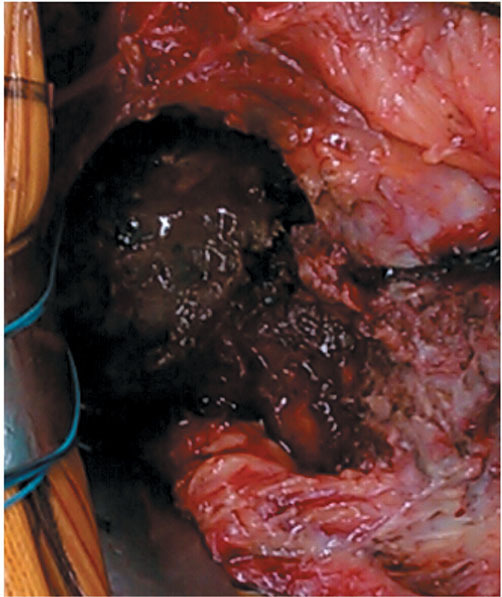
Upon incision of the joint capsule, a dark brown muddy substance spouts from inside the joint. When dislocated, the femoral head and neck are colored dark brown.

**Figure 6.**
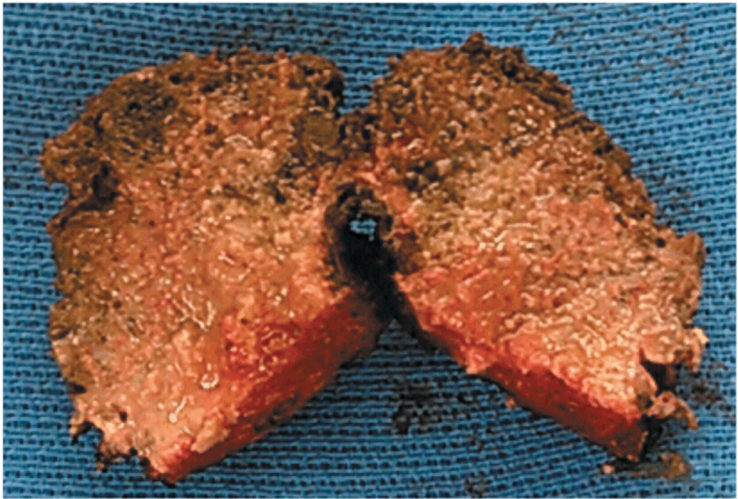
A fractured section of the femoral head showing that the pigmentation extends into the bone.

Right-side total hip arthroplasty (THA) was performed for rapidly destructive hip arthropathy due to alkaptonuria (duration: 196 min; blood loss: 830 mL). Caution was required to avoid excessive acetabular reaming due to severe osteoporosis, and bone cement was used considering the osteoporotic bone quality. Dark brown and muddy contents were observed in the joint capsule. Macroscopic findings of the excised femoral head showed a destroyed weight-bearing surface with dark brown pigmentation (Figure 5), while there were map-like black pigmentations on the acetabular surface. Moreover, a proliferated synovium was identified. On the fractured surface of the femoral head, the black tissue infiltrated the bone from the articular surface (Figure 6). 

**Figure 7.**
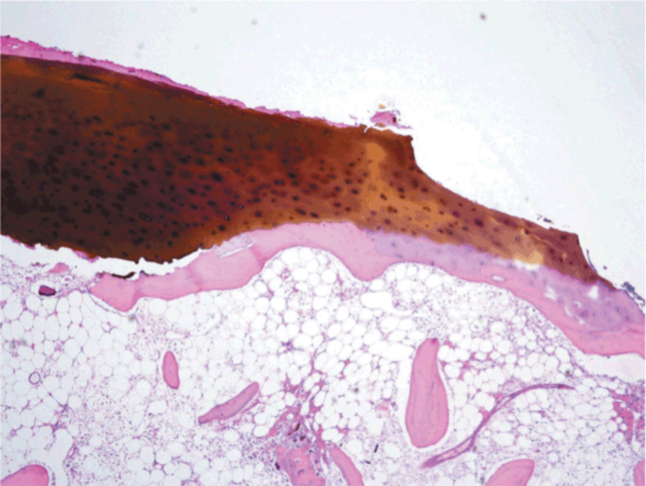
Histopathological findings show pigmented cartilagenous tissue (hematoxylin and eosin staining × 100) with thinner and lower density trabecula beneath the cartilage tissue.

**Figure 8.**
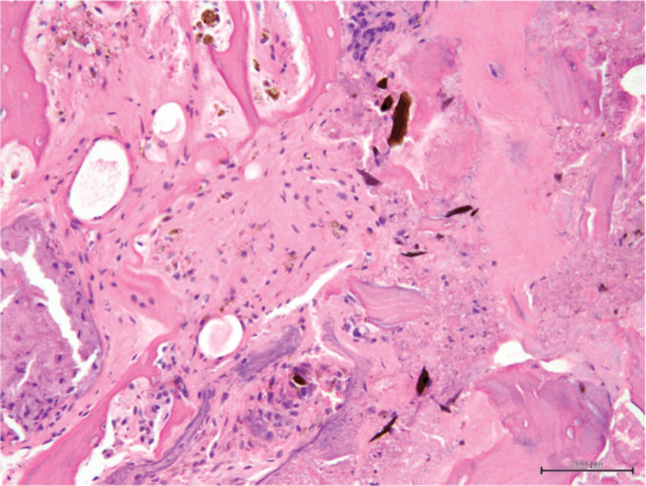
Histopathological findings also show the granulomatous lesions in the medullary cavity and invasion of the destroyed cartilage (hematoxylin and eosin staining × 200).

Microscopy revealed a brownish tint of the partially remaining cartilage (Figure 7) and granulomatous lesions containing fragmented bone and cartilage debris were observed in the medullary cavity. There was no evidence of osteonecrosis (Figure 8). 

After THA, the patient commenced full weight-bearing walking, which gradually became painless, after which he could undertake more activity than before. Eight months after surgery, his modified Harris hip score was 84. Written informed consent was obtained from the patient to publish the report.

## Discussion

There is no radically effective treatment for alkaptonuria. To reduce various symptoms due to HGA accumulation by suppressing HGA production, nitisinone is considered as a new therapeutic agent for alkaptonuria because it is an inhibitor of 4-hydroxyphenylpyruvate dioxygenase, which is an HGA-producing enzyme.^5^ Patients with alkaptonuria that are >40 years of age are known to develop cardiovascular complications, such as aortic dilatation, aortic or mitral regurgitation, and urinary complications, such as prostate and kidney stones. However, the prognosis for survival in patients with alkaptonuria is generally good. Therefore, symptomatic treatment is important for alkaptonuria. For locomotive disorders that are more likely to develop from the age of 20, physical therapy is usually given to maintain muscle strength and flexibility; pain control is achieved with appropriate administration of analgesics; and THA is often performed in patients with advanced joint destruction.

Joint destruction in alkaptonuria is attributed to organic changes caused by the deposition of HGA on the surfaces of cartilage tissues, which reduces the elasticity of the cartilage. Subsequently, mechanical stress damages the cartilage and subchondral bone.^3,6^ There are some reports of such cartilage disorders. For example, Gil et al. reported a case of joint space narrowing that occurred early in the development of the disease, similar to coxarthrosis.^3^ Furthermore, there are many reports of knee joints with arthropathy findings that originate from cartilage disorders.^7^ Conversely, there are several reports of bone loss due to alkaptonuria,^8-^^10^ and one report showed that five among seven patients with alkaptonuria, excluding the youngest two patients, had osteopenia.^10^ The same study found an increase in receptor activator of nuclear factor-kappa Β ligand and a decrease in osteoprotegerin and that the activation of osteoclasts due to this increase and decrease is responsible for bone loss in patients with alkaptonuria.^11^ Similarly, in our patient, the bone resorption marker (TRACP-5b) and bone permeability were increased, and the bone mineral density of the femoral neck was significantly reduced. Thus, increasing mechanical stress to the subchondral bone with reduced cartilage elasticity should increase the risk of subchondral fracture due to concurrently progressing osteoporosis.

In 1957, Forestier^12^ first described a rare syndrome of unknown etiology that caused rapid destruction of both the femur and acetabulum of the hip joint, leading to the femoral head disappearing almost completely within a few months. In 1970, Postel and Kerboull^4^ reported a disease in which a hip joint with a normal spherical femoral head, such as in rheumatoid arthritis, was characterized by the destruction of the femoral head within 6 months to 1 year, and considered as a rapidly destructive hip disease. One of its causes is subchondral insufficiency fracture (SIF) of the femoral head due to bone fragility associated with osteoporosis.^11^ Hamada et al. reported a case of SIF of the femoral head with alkaptonuria.^13^ Accordingly, in our patient, within approximately 3 months from onset, the acetabulum showed almost no bone defects, although half of the femoral head was destroyed—evidence of acute destructive hip disease. Pathological images showed minor necrosis due to destruction but no femoral head necrosis. Furthermore, granulomatous lesions were mainly found on the cartilage tissue and bone fragments that had invaded the bone marrow, which is characteristic of rapidly destructive hip disease and similar to the histopathological image of RDC. Since our patient had marked osteoporosis, it can be inferred that rapidly destructive hip disease was caused by SIF of the femoral head due to increased mechanical stress. Deposition of HGA infiltrated into the bone from the surface; however, it was unclear whether HGA caused bone destruction directly after sustaining SIF. Although joint destruction in alkaptonuria is reportedly caused by organic changes in cartilage tissue due to HGA deposition, there may be cases of osteoporosis-based SIF leading to RDC that can be traced to underlying disease. Since the patient had alkaptonuria, it did not strictly adhere to the definition of RDC as proposed by Postel and Kerboull. Review of published case reports revealed insufficient imaging and histopathological findings showing typical RDC with destructive bone defect of the femoral head in a short period, although a few patients with alkaptonuria appeared to have RDC.^14,15^ The present report is the first to reveal RDC in alkaptonuria using detailed imaging and histopathological findings.

We presented a case of rapidly destructive hip disease due to alkaptonuria, suggesting an RDC due to bone fragility and cartilage degeneration due to HGA deposition. Alkaptonuria patients require early therapeutic intervention, such as treatment for osteoporosis to prevent rapidly destructive hip disease.

**Highlights**

- Despite there are earlier reports on rapidly destructive coxopathy associated with alkaptonuria, this is the first case report that has appropriately shown this pathology using imaging and histopathological findings.
- We found granulomatous foci containing fragmented bone and cartilage debris in the bone marrow space of the joint surface and brownish-black–pigmented cartilage tissue; ochronotic hip joint destruction was diagnosed.
- This is the first report of rapidly destructive coxopathy associated with alkaptonuria, appropriately shown using imaging and histopathological findings.

## Supplementary Data

Supplementary Figure S1
