# Supplementary figures and images for: Alkaptonuria with rapidly destructive arthropathy of the hip: A case report and literature review
Source: Acta Orthop Traumatol Turc. 2021 Nov 1;55(6):563–9. doi: 10.5152/j.aott.2021.21205 (PMC11583233; doi:10.5152/j.aott.2021.21205)

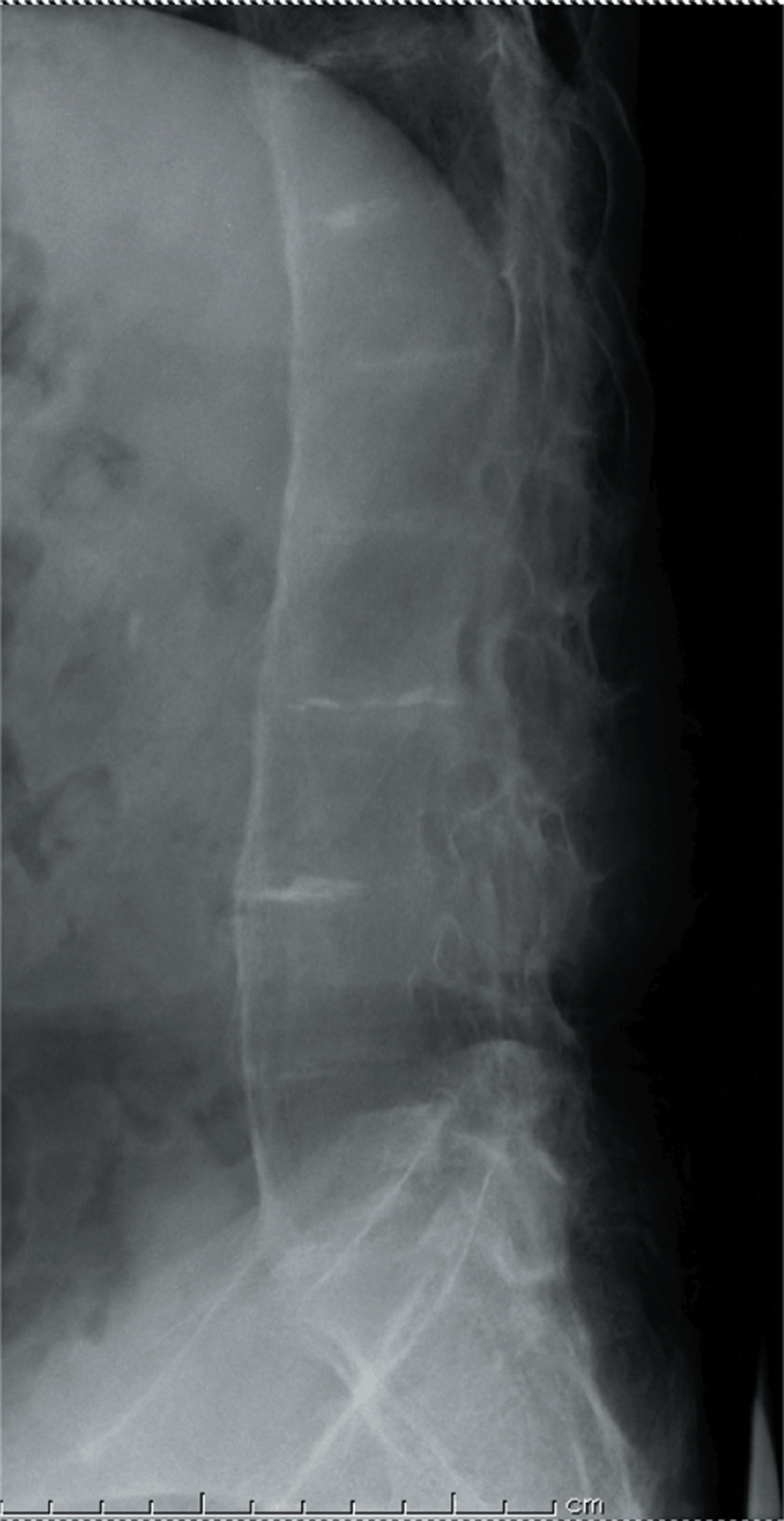

Supplement: Supplementary Figure S1 [file AOTT-D-21-00205-ug01.jpg]
